# Co-Morbidity of *DSM-IV* Personality Disorder in Major Depressive Disorder Among Psychiatric Outpatients in China: A Further Analysis of an Epidemiologic Survey in a Clinical Population

**DOI:** 10.3389/fpsyt.2019.00833

**Published:** 2019-11-12

**Authors:** Yuchen Zheng, Francesca Severino, Li Hui, HaiSu Wu, Jijun Wang, Tianhong Zhang

**Affiliations:** ^1^Shanghai Mental Health Center, Shanghai Jiao Tong University School of Medicine, Shanghai, China; ^2^Department of Public Health, Laboratory for Mother and Child Health, Mario Negri Institute for Pharmacological Research, Milan, Italy; ^3^Institute of Mental Health, Suzhou Psychiatric Hospital, The Affiliated Guangji Hospital of Soochow University, Suzhou, Chinaz; ^4 ^Shanghai Mental Health Center, Shanghai Jiaotong University School of Medicine, Shanghai Key Laboratory of Psychotic Disorders, Shanghai, China

**Keywords:** personality disorder, major depressive disorder, anxiety disorders, co-morbidity, China

## Abstract

**Introduction:** It is common that personality disorder (PD) co-occurs with major depressive disorder (MDD). In the current literature, there is a dearth of information on the co-occurrence of PD and MDD among Chinese population.

**Materials and Methods:** 609 individuals were randomly sampled from outpatients diagnosed as MDD in Shanghai Mental Health Center. Co-morbidity of PDs was assessed using the Personality Diagnostic Questionnaire Fourth Edition Plus (PDQ-4+) and eligible subjects were interviewed with the Structured Clinical Interview for *DSM-IV* Axis II (SCID-II). The score of PDQ-4+ and the rate of SCID-II PD between subjects diagnosed with MDD and those with anxiety disorders (AD) were compared.

**Results:** Two hundred fifty-eight outpatients (42.36%) with MDD were recognized to possess at least one criterion of diagnosis for PD, according to the *DSM-IV*. The most prevalent PD was depressive PD (14.61%), followed by avoidant (11.49%) and borderline (11.49%) PD. Cluster C PDs (anxious and panic PD) were the most common PD types (12.12%) when compared to other clusters. Compared to patients with AD, individuals with MDD were significantly more likely to have paranoid PD (6.6% vs. 3.3%, p = 0.011), borderline PD (11.5% vs. 3.7%, p = 0.000), passive-aggressive PD (5.6% vs. 2.4%, p = 0.007), and depressive PD (14.6% vs. 7.8%, p = 0.000).

**Discussion:** The finding indicates that there is a high prevalence of PD among patients with MDD. More significant co-morbidity rates of PDs in MDD have been found when compared with AD. Further studies for the longitudinal impact of the PD-MDD co-morbidity are in need.

## Introduction

Personality disorder (PD) with major depressive disorder (MDD) has been one of the most common co-morbidities in mental health disorders (1, 2). According to former studies, up to 3/4 of patients with PD have suffered from at least one episode of MDD (3, 4). Understanding the relation between PD and MDD has clinical importance in prognostic accuracy and individualized interventions of depression, with the fact that the co-morbidity would complicate the treatment and worsen the prognosis of this disorder (5, 6). It has been generally recognized that the chronicity of depression is related with a longer duration of untreated illness (DUI) (7), which might be influenced by many indicators (8). This underlines the necessity of considering the impact of the influential factors with poorer prognosis and incorporate specific interventions toward PD, including pharmacological (9–11), psychological (12–16), and social interventions (17).

Till now, many former studies have collected epidemiological data on the co-morbidity of PD and MDD (18–22). The reported prevalence of PD in MDD vary among previous studies, with the average rate of co-morbidity being approximately 50% (18, 21–23). The most frequently co-occurred PD subtypes were in turn: avoidant, borderline, dependent, obsessive-compulsive (24), and self-defeating PDs (1).

Although previous studies have been discussing the relationship between personality and depressive disorders in the past—with contributions ranging from the fields of epidemiology, epigenetics, and clinical treatments—the results have shown variations across countries, possibly due to the influence of different socio-cultural contexts (3, 4, 25–27). Moreover, there is a relative dearth of information in the current literature of Chinese patients with MDD. The only piece of Chinese literature accounting for pathological personality in MDD was published a decade ago, with a small sample of 102 patients with depressive disorders diagnosed by structured clinical interview for *DSM-IV* (SCID-II) (28). Considering the substantial difference between Eastern and Western culture, as well as the rapid changes in Chinese society (including the influence of westernization and modernization in the latest decades), the study in Chinese samples could not only guide contextually grounded clinical work with MDD patients with PDs but would also provide a comparable data to further understanding the interaction of the two disorders.

Research focusing on PDs among Chinese samples started in the 1980s (29, 30). Nevertheless, with the limitation of the Chinese diagnostic system, the prevalence rates of PD in the general Chinese population were underestimated (0.1‰–0.13‰). In 2000, Yang et al. has reported the prevalence of *DSM-IV* PDs in a sample of Chinese psychiatric patients, supporting the high clinical prevalence rates in Chinese population (31). In our former studies, a large-scale survey has been carried out and results led to the conclusions that the co-morbidity rate of PD with mood disorder was 42%, which is the highest among all the conditions (schizophrenia, mood disorder, and anxiety disorder [AD]) (32, 33). However, the demographic and clinical profile of the co-morbidity between PD and MDD is still unclear to the present date. The primary objective of this study is to examine the distribution of PDs in Chinese patients with MDD, using both *DSM-IV* (clinical interview) and the PDQ-4+ (self- report questionnaire) to assess personality traits. Anxiety-related disorders were settled as a comparative group to examine the difference in disturbance of PD subtypes. The possible intervention strategies of recognizing or treating specific pathological personality subtype in China would also be discussed in this study.

## Materials and Methods

### Sample Characteristics

The epidemiologic survey was conducted in one of the largest medical health service settings in China, the Shanghai Mental Health Center, in 2006 (32–37). As what has been comprehensively described in former studies (32, 33, 35), the participants were randomly sampled from the psycho-counseling and psychiatric clinics at the same hospital. To be briefly reviewed, a total of 3,402 individuals were randomly sampled between May and October 2006. Invitation letters were sent to them with a self-report questionnaire to collect information about PD.

The exclusion criteria were as follows: (a) presence of serious or acute psychotic symptoms; (b) a documented organic mental disorder; and (c) mental retardation. According to the exclusion criteria, subjects with serious or acute psychotic symptoms were excluded, ensuring that the participants included were in a stable state and with a degree of insight. Three hundred twenty-seven (9.6%) ineligible individuals were excluded. The overall response rate was 90.4%. More description of the inclusion and exclusion criteria could be found in our previous publications (32–34).

Out of the 3,075 patients included for PD assessment, 609 outpatients (19.8%) were diagnosed with MDD. Amongst the 609 patients (214 men, 395 women), with a mean age of 33.19 years (SD = 10.4, ranged 18–60 years), 359 of them (58.9%) were sampled from the psycho-counseling clinic and 250 individuals (41.1%) were recruited from the psychiatric clinic.

Three hundred seventy-five patients (61.6%) came to the clinic for the first time, whereas 111 patients (18.2%) showed at the location repeatedly (more than five times before the study). Eighty-seven patients (14.3%) have received pharmacological treatments, including antidepressant drug (n = 73), antipsychotic drug (n = 7), hypnotic drug (n = 4), and anxiolytics drug (n = 3). Seventy-seven patients (12.4%) had medical comorbidity, including cardiovascular disease (n = 15), digestive disease (n = 20), endocrinology disease (n = 8), immunology disease (n = 5), body pain (n = 8), tumor (n = 5), insomnia (n = 3), other diseases (n = 11), and multiple diseases (n = 2). The length of disorder varied from 0 to 372 months, with a mean course of 43 months. When taking marital status into consideration, 248 participants (40.7%) were single, 303 (49.8%) were married, 36 (5.9%) were divorced, 22 (3.6%) were widowed. As for the educational level, 316 (51.9%) participants were graduates, while only 73 (12.0%) participants earned more than 5,000 Yuan a month. Ninety (14.8%) participants had not been raised by both parents.

To make a comparison with MDD patients, 574 participants who were diagnosed with anxiety and related disorders were selected from the sample of 3,075 outpatients according to their medical records. Among these patients, more than a half of them were diagnosed with a general AD and panic disorder (n = 343), 153 with obsessive-compulsive disorder, 78 of them with phobia. The average age of the 574 patients (278 men, 296 women) was 32.52 years (SD = 10.0, ranged 18–60 years). ANOVA and Chi-squared tests were used to compare the results of PD assessment by using PDQ-4+ and SCID-II separately among different ADs (see Supplement). The results of most of PD subtypes, except schizotypal, avoidant, and obsessive-compulsive PD, did not present significant differences among these ADs.

### Measures

#### General Questionnaire

The general questionnaire collected data that includes: (a) demographics; (b) family and social background; and (c) physical and mental health conditions.

#### Assessment of Personality Disorders

The Personality Diagnostic Questionnaire Fourth Edition Plus (PDQ-4+): as detailed in our previous studies (32–35), the PDQ4+ is a concise structured self-report questionnaire that contains 107 true-false items and screens for 12 Axis II *DSM-IV* Personality Disorders. The PDQ-4+ seeks to discriminate between individuals with and without characteristics that can be associated with PD (38–40). Although the specificity is medium (.65), the PDQ-4+ is a highly sensitive test (.89). It has been used to screen for *DSM-IV* PD in the Chinese psychiatric patients (31, 32) and college student populations (41). The test-retest reliability value amongst the Chinese populations was of .92.

The Structured Clinical Interview for *DSM-IV* Axis II (SCID-II): Is a Semi-Structured Clinical Interview for PD Diagnosis. the Pds Classification Is Made in Accordance With the *DSM-IV* Criteria, Which Includes Cluster a PD (Paranoid, Schizoid, Schizotypal PD), Cluster B PD (Histrionic, Narcissistic, Borderline, Antisocial PD), Cluster C PD (Avoidant, Dependent, Obsessive-Compulsive PD), Passive-Aggressive PD and Depressive PD (In the Appendix of *DSM-IV*). the SCID-II Is Consistent (.90) With the Clinical Diagnosis and Has a Relatively High Test-Retest Reliability (.70), With a Median of Coefficient for Internal Consistency Of.70 (42, 43).

#### Diagnosis of Major Depressive Disorder and Other Axis I Disorder

Patients' clinical diagnoses were assessed with the Chinese Classification and Diagnostic Criteria of Mental Disorders version 3, CCMD-3 (Chinese Society of Psychiatry, 2001) by psychiatrists at outpatient appointments. The CCMD-3 represents a classification of mental health disorders substantially influenced by *ICD-10* and *DSM-IV* representations. Most of the diagnostic criteria of MDD and AD (including phobia, panic disorder, general AD, obsession) are identical to the international classifications.

### Procedures

The Research Ethics Committee at the Shanghai Mental Health Centre approved the study in 2006. The sample was selected within a two-stage procedure. Firstly, 3,402 individuals were randomly sampled from the psycho-counseling and psychiatric clinics internal to the Shanghai Mental Health Center and invited to participate in the study. The participants were asked to complete the general questionnaire and PDQ-4+, under the guidance of three senior executive nurses. The data were checked by the trained nurses, to ensure that each questionnaire was completed. Then the PDQ-4+ was administered by a trained psychiatrist to screen for the presence of PD. Amongst the 3,075 eligible individuals, 2,706 patients met the criteria for *DSM-IV* PD and were recruited into the second stage of the study, the SCID-II interview.

In order to reduce the effect that subjective deviation had on the results of the SCID-II clinical interview, the interviewers, two senior psychiatrists who were trained for 2 weeks by the research team members (ZP, Xiao; YF, Dai et al.), were not aware of the PDQ-4+ test results and clinical diagnoses of the patients. The inter-rater reliability of the SCID interview was determined after training. Thirty inpatient interviews were scored by two raters independently. The kappa value of reliability for any PD was 0.82.

All participants were diagnosed according to CCMD-3 by the attending psychiatrists, who were unaware of the results of the personality evaluation of the patients. The clinical diagnoses of participants reported in this paper were made by two attending psychiatrists separately and written down in their outpatients' medical records, which was double-checked by researchers. In this study, 609 medical records of patients with MDD were selected from the large sample of outpatients. Among them, 536 were PDQ-4+ positive and interviewed with SCID-II. The interviewer reliability for PDQ4+ and SCID-II was satisfactory. The reliability of raters, according to the kappa value for the diagnosis of PD, fell between.78 and.98.

### Statistical Analysis

SPSS 20.0 (SPSS Inc., Chicago, IL, USA) was used to analyze participants' demographic and clinical data. Frequencies and 95% confidence interval (95% CI) of PDs in patients with MDD, evaluated by PDQ-4+ and SCID-II, were calculated separately by cluster and specific PD. Odds ratios (OR) were generated to assess associations of PDs with demographic and clinical profiles such as age, gender, education and marriage state, parents' marriage state, type of outpatient service (psycho-counseling or psychiatric clinic), and self-reported characteristics. Two tailed t-tests were used to compare the average score of PDQ-4+ by cluster and specific PD between MDD and AD, while Chi-squared tests were used to compare the prevalence of PD detected by SCID-II. Stepwise regression was performed using clinical diagnosis (MDD vs. anxiety and related disorders) as dependent variables, with social-demographic characteristics (age, gender, marital status, parental divorce, normal trait, caregiver) and each cluster of PD as independent variables. All statistical differences were considered significant at p < .05.

## Results

### Prevalence of PDs

**Table 1** reflects the prevalence of PDs among patients with MDD. According to the results of PDQ-4+, the rate of self-reported PDs in the depression sample was relatively high: 88.01% of the patients presented at least one self-reported PDs. The most frequent PD in this sample was avoidant PD (62.4%), followed by obsessive-compulsive (61.1%) and borderline PD (BPD) (54.02%). In addition, the structured interviewing tool of SCID-II was used to investigate clinical diagnosed PDs. The results significantly showed the prevalence of PD among patients with MDD with lower occurrence rates than the PDQ-4+ results. Among individuals 42.36% met at least one criterion for *DSM-IV* PD. The most prevalent PD was depressive (14.61%), followed by avoidant (11.49%) and BPD (11.49%). Cluster C PDs (12.12%) were the most common PD types compared to other clusters.

**Table 1:** Frequency of *DSM-IV* personality disorders (PDs) among patients with depression using Personality Diagnostic Questionnaire Fourth Edition Plus (PDQ-4+) and Structured Clinical Interview for *DSM-IV* Axis II (SCID-II).

	PDQ-4+		SCID-II	
	n (%)	95% CI	n (%)	95% CI
Any PDs	536 (88.01)	85.43–90.60	258 (42.36)	38.43–46.30
Any PDs (appendix excluded)	531(87.2)	84.53–89.85	227 (37.27)	33.42–41.13
Any Cluster A PD	383 (62.89)	58.91–66.62	65 (10.54)	8.09–12.99
Paranoid PD	304 (49.92)	45.94–53.90	40 (6.57)	4.60–8.54
Schizoid PD	162 (26.6)	23.08–30.12	10 (1.64)	0.63–2.65
Schizotypal PD	219 (35.96)	32.14–39.78	19 (3.12)	1.74–4.50
Any Cluster B PD	406 (66.67)	62.96–70.48	93 (15.32)	12.45–18.19
Histrionic PD	227 (37.27)	33.42–41.13	15 (2.46)	1.23–3.70
Narcissistic PD	210 (34.48)	30.70–38.27	15 (2.46)	1.23–3.70
Borderline PD	329 (54.02)	50.05–57.99	70 (11.49)	8.95–14.03
Antisocial PD	150 (24.63)	21.20–28.06	1 (0.16)	−0.16 to 0.49
Any cluster C PD	473 (77.67)	74.27–80.92	138 (22.41)	19.08–25.73
Avoidant PD	380 (62.40)	58.54–66.26	10 (11.49)	8.95–14.03
Dependent PD	226 (37.11)	33.26–40.96	19 (3.12)	1.74–4.50
Obsessive-compulsive PD	372 (61.08)	57.20–64.97	60 (9.85)	7.48–12.23
In the appendix of *DSM-IV*	363 (59.61)	55.56–63.39	113 (18.62)	15.51–21.72
Passive-aggressive PD	260 (42.69)	38.75–46.63	34 (5.58)	3.75–7.41
Depressive PD	289 (47.45)	43.48–51.43	89 (14.61)	11.8–17.43

### Impact of Clinical and Socio-Demographic Characteristics

The overall prevalence of PDs among young people was significantly greater than the prevalence in older people (OR = 1.343) (**Table 2**). Compared with the group of married individuals, patients who were single (OR = 1.352) had a higher prevalence of PD. The prevalence of PD was greater in the psycho-counseling clinic (OR = 1.144) than in psychiatric clinic. Patients whose parents were divorced (OR = 1.663) were more likely to be diagnosed with PD. On the other hand, patients who were raised by their parents (OR = 0.627) were less likely to get diagnosed with PD. The prevalence of PD was higher in individuals with Introversion characteristics (OR = 1.348) than those with other types of characteristics (middle type, extraversion).

**Table 2:** Odds ratios (and 95% CI) of having Structured Clinical Interview for *DSM-IV* Axis II personality disorders (PDs) among patients with depression, by clinical and socio-demographic characteristics.

	Number	PD	PD (%)	Odds ratio	95% CI
∼30 years	312	155	49.68	1.343	1.153–1.565
30∼ years	297	103	34.68	0.722	0.605–0.862
Single or divorced, widowhood	305	152	49.84	1.352	1.156–1.581
Married	304	106	34.87	0.728	0.613–0.866
Parents divorced	60	33	55	1.663	1.026–2.694
Parents together	549	225	40.98	0.945	0.894–0.999
Psycho-counseling clinic	359	164	45.68	1.144	1.003–1.305
Psychiatric clinic	250	94	37.6	0.820	0.672–1.000
Raised by parents	519	211	40.66	0.932	0.869–0.999
Raised by others	90	47	52.22	1.487	1.016–2.177
Introversion	213	106	49.77	1.348	1.087–1.671
Middle type	274	107	39.05	0.872	0.727–1.046
Extraversion	122	45	36.89	0.795	0.571–1.107

Age grouped by median age of the sample.

### Comparative Analyses of Personality Disturbance

**Table 3** has shown the personality disturbance between MDD and AD, evaluated by PDQ-4+ and SCID-II. According to the results in the first set of analyses, individuals with MDD reported significantly more symptoms of all PDs compared to individuals with AD, except for paranoid and schizotypal PD. In the second set of analyses, which was conducted to investigate the difference of PD prevalence between the MDD and AD group, individuals with MDD were significantly more likely to have paranoid PD (6.6% vs. 3.3%, p = 0.011), BPD (11.5% vs. 3.7%, p = 0.000), passive-aggressive PD (5.6% vs. 2.4%, p = 0.007), and depressive PD (14.6% vs. 7.8%, p = 0.000).

**Table 3:** Comparison of difference between major depressive disorder (MDD) and anxiety disorders for Personality Diagnostic Questionnaire Fourth Edition Plus (PDQ-4+) personality disorders (PDs) scores and Structured Clinical Interview for *DSM-IV* Axis II (SCID-II) PDs frequency.

	PDQ-4+			SCID-II (%)		
	MDD	AD	t value	MDD	AD	*χ* ^2^ value
Any Cluster A PD	9.34	8.74	2.253*	10.7	6.8	0.023*
Paranoid PD	3.03	2.91	1.156	6.6	3.3	0.011*
Schizoid PD	2.55	2.27	2.997**	3.1	1.9	0.201
Schizotypal PD	3.75	3.56	1.480	1.6	2.1	0.668
Any Cluster B PD	14.02	12.13	5.023**	15.3	9.4	0.003**
Histrionic PD	3.79	3.48	2.880**	2.5	4.2	0.105
Narcissistic PD	3.65	3.15	4.327**	2.5	3.0	0.721
Borderline PD	4.82	4.09	4.092**	11.5	3.7	0.000**
Antisocial PD	1.76	1.41	3.582**	0.2	0.7	0.206
Any Cluster C PD	11.87	10.75	3.896**	22.7	21.8	0.727
Avoidant PD	4.16	3.71	3.972**	11.5	9.1	0.181
Dependent PD	3.63	3.24	3.106**	3.1	2.8	0.864
Obsessive-compulsive PD	4.08	3.81	2.446*	9.9	12.7	0.140
In the appendix of *DSM-IV*	7.23	6.52	3.764**	18.6	9.2	0.000**
Passive-aggressive PD	3.09	2.83	2.728**	5.6	2.4	0.007**
Depressive PD	4.14	3.70	3.785**	14.6	7.8	0.000**

Levene's test for equality of variances is significant; *p < 0.05; **p < 0.01.

### Stepwise Regression Analyses

Stepwise regression was employed in an attempt to identify the risk factors of PDs related to MDD or AD, evaluated with PDQ-4+ and SCID-II. Logistic regression (forward stepwise) analyses were performed with clinical diagnosis (MDD vs. AD) as the dependent variable while social-demographic characteristics (age, gender, marital status, parental divorce, normal trait, caregiver) and different type of PDs acted as independent variables (**Table 4**). The results from self-report questionnaire (**Table 4**) indicated that borderline and narcissist PDs were significant predictors of MDD, while paranoid PD was a significant predictor of AD. The results evaluated by interviews showed that borderline and depressive PDs were significant predictors of MDD.

**Table 4:** Forward stepwise logistic regression for risk factors predicting the clinical diagnoses based on Structured Clinical Interview for *DSM-IV* Axis II (SCID-II) and Personality Diagnostic Questionnaire Fourth Edition Plus (PDQ-4+).

MDD vs. ADs	Variable	beta	SE	Odds ratio	95% CI	*χ* ^2^ statistic	*p* value
SCID-II	Gender	0.544	0.122	1.723	1.356–2.190	19.784	0.000
	Parent's marriage	0.463	0.222	1.589	1.028–2.455	4.347	0.037
	Borderline	1.021	0.267	2.775	1.645–4.682	14.624	0.000
	Histrionic	−0.847	0.352	0.429	0.215–0.854	5.803	0.016
	Depressive	0.547	0.202	1.728	1.162–2.570	7.294	0.007
	Constant	−0.945	0.205	0.389	—	21.285	0.000
PDQ-4+	Gender	0.533	0.121	1.704	1.410–2.242	19.390	0.000
	Borderline	0.128	0.043	1.137	1.074–1.230	16.347	0.000
	Narcissistic	0.093	0.037	1.098	1.012–1.169	6.355	0.012
	Paranoid	−0.122	0.043	0.885	0.823–0.976	8.137	0.004
	Constant	−1.311	0.234	0.269	—	31.310	0.000

## Discussion

The data gathered from this study has filled the existing gap in literature about the co-morbidity of PD-MDD in Chinese population. The primary aim of this study was to examine the distribution of PDs in Chinese patients with MDD. The result of this study indicates that the co-morbidity of PD was indeed common among Chinese patients with MDD: 88.01% of participants were presenting at least one self-reported PD, and 42.36% patients with MDD were meeting the clinical criteria of PD. According to former epidemiological studies, the number of rate of co-morbidity came within the average being approximately 50% (18). However, considering that the varied prevalence in previous surveys may have been influenced by different sample characteristics and research methods, comparing the rates with foreign studies without proper adaptive measures would represent a problem. Particularly, careful consideration is needed when taking timing of assessment, source of information, and diagnostic instruments into account (44).

Among three clusters of PDs in MDD patients, Cluster C PDs were the most common types, while Cluster A PDs were the least frequent. The possible explanation might be that Cluster C PDs are predominantly treatment-seeking unlike the other two types (45). Moreover, the help-seeking behaviors declined in Cluster A PDs as the results of stigma and social disability, which might in turn be caused by the lower prevalence of this cluster among the other three clusters. The distribution of different PD subtypes in MDD patients was mostly in accordance with the results in previous studies (24, 27), suggesting that avoidant and BPDs were two most common PD subtypes in patients with MDD.

The socio-demographic characteristics of MDD patients with PD have also been investigated in this study. The factors associated with increased odds of PD were shown to include a younger age, non-marriage status (single, divorced, or widowhood), not being raised by both parents, belonging to a family with divorce history, being in psycho-counseling clinic, and scoring higher as introverted in self-reported measures. This has replicated the finding in previous studies such as the Australian National Survey of Mental Health and Wellbeing, which led to the conclusion that youths and not married individuals were also more likely to have PD (46–49). The effect of age on PD prevalence has been supported by some studies (36, 50) but rejected by others (46, 51, 52). Detailed discussion could be found in our former study (36).The other result of these factors calls for needs to build a stronger supporting system as a protective measure.

The result of the comparison of PDs between MDD and AD was unexpected. When compared with Previous literature reported that PD patients were five times more likely to have an AD and four times more likely to have a mood disorder (46). However, in our studies, the prevalence of PD among patients with AD (84.8% with PDQ-4+ and 34.3% with SCID-II) turned out to be lower than the prevalence among patients with MDD. The comparably greater rates of PD among MDD patients was also supported by the distinct high co-morbidity of "eccentric" and "emotional" group PDs when compared to the patients with AD, while there was no significant difference in the "fearful" group of PDs. The higher rate of PD occurrence reflected by the self-report questionnaire, compared to the result of the clinical interview, possibly indicated the negative cognition of MDD patients to their own situation (53). Zimmerman et al. argued that diagnoses based on self-reported questionnaires might be more sensitive to clinical state than clinical interviews are (54). These different results were caused by variation in participant responses across measures. Shannon et al. found that participants with lower severity of disorder may show greater disclosure on anonymous inventories relative to interview formats (55). It might be because of lower defensiveness and fuller reporting of symptoms in the more anonymous paper and pencil setting of a self-report inventory (31). The difference in diagnoses of PD by using self-report questionnaires and clinical interviews requires further studies.

Based on the results of predictive factors, borderline and depressive PDs seemed to be strongly associated with MDD, while Histrionic PD was predictive of AD. Conceptualized as diagnostically distinct disorders, MDD and AD have high co-morbidity (56, 57), thus, more understanding about these two disorders are in need. This result of this study might provide specific clinical clues in understanding the personality characteristic between depression and ADs. The possible reasons for the inter-relationship of PDs and Axis I disorders has been discussed in our former studies (37), including the pre-morbid model and the post-morbid model. Meanwhile, Johnson et al. proposed that some PDs and mood disorders might be at different points on a common affective spectrum (58). The co-occurrence could also be partly attributable to overlapping diagnostic criteria between the two disorders.

The result of the stepwise logistic regression is consistent with previous studies (3, 59, 60), confirming that the BPD is the most significant predictor of MDD. Specifically, Gunderson et al. have found that among different types of PDs, BPD patients were most likely to have MDD and 85% of the patients with co-morbidity had more than one recurrence of a depressive episode (3, 59) Reichborn-Kjennerud et al. have paralleled this result with a twin study of 2,801 patients, which indicates that borderline, avoidant, and paranoid PDs could increase the risk of MDD and that, once again BPD was the strongest predictor of MDD (60).

The result of high prevalence and correlation with MDD of depressive PD, a PD subtype in *DSM-IV* appendix and now removed from the *DSM-5* category, was parallel to previous studies, supporting that depressive PD is one of the most commonly diagnosed personality subtypes (61) and highly overlaps with MDD (62). Although this diagnoses was no longer included in current classification systems of mental disorders, partly because it overlaps with other diagnoses (63), there're still some arguments that depressive PD should be included as a diagnostic type (64, 65). Considered of these reasons, the result of depressive PD was retained in the analysis and might provide further evidence to future studies.

It is important to note that there are several methodological limitations in this study. Firstly, as a cross-sectional survey, it is unable to investigate the order of occurrence between PD and MDD. Meanwhile, some patients with a first depressive episode might subsequently change their diagnosis in bipolar disorder (66), the number of which was unclear under such limitation. To evaluate the longitudinal relationship of PD and MDD, as well as to minimize the influence of misdiagnosis, prospective follow-up studies of PD and MDD are in need. Secondly, the sample of this study was made up from outpatients from psycho-counseling and psychiatric clinics in one hospital in Shanghai. Hence, the results might not be generalized to a broader population with MDD in China. Thirdly, the prevalence of PDs based on SCID-II might be underestimated by using the PDQ-4+ as a screen. In this study, only patients with positive results on the PDQ-4+ entered the clinical interview with SCID-II. Although this self-report questionnaire has a high sensitivity (0.89), some participants with false negative results might be excluded. Moreover, the sensitivity of the PDQ-4+ varies by diagnosis (31, 32), which could also impact the diagnoses on different levels. Lastly, only one major diagnosis of the patients was collected (when some may have had two or more), which might not take the influence of other co-morbidity into consideration. Such influence could be bidirectional. The overlaps between some of PD symptoms and other Axis I disorders, such as schizophrenia and obsessive-compulsive disorder, may affect the accuracy of personality assessment and lead to an overestimation of PD prevalence. Meanwhile, the exclusion of some Axis I disorders, such as substance use disorders, which have high co-morbidity rate with PD (67, 68), might lead to an underestimation of the PD rates among the participants.

## Conclusion

In summary, this study has provided important clues to understanding the relationship between MDD and PD and has highlighted the assessments and interventions of PD in patients with MDD. The results suggested that indicators, such as specific PD subtypes, might be helpful in further understanding how PD correlates to the difference between MDD and ADs. Further studies for longitudinal impact of the co-morbidity, including the psychopathology, progression, and outcome, could further reveal useful information on this topic.

## Data Availability Statement

The datasets generated for this study are available on request to the corresponding author.

## Ethics Statement

This study was carried out in accordance with the recommendations of the Research Ethics Committee at the Shanghai Mental Health Centre with written informed consent from all subjects. All subjects gave written informed consent in accordance with the Declaration of Helsinki. The protocol was approved by the Research Ethics Committee at the Shanghai Mental Health Centre.

## Author Contributions

YZ designed and conducted this study, including data analysis and writing the article. FS revised the article. LH was responsible for collecting and entering data. HW and JW were responsible for recruiting, diagnosing, and classifying the patients. TZ revised the study design and the article.

## Funding

This work was supported by the Ministry of Science and Technology of China, National Key R&D Program of China (2016YFC1306803), National Natural Science Foundation of China (81671329, 81671332), Clinical Research Plan of SHDC (16CR2015A, 16CR3016A), Shanghai Mental Health Center Foundation (2016-FX-01, 2017-TSXK-03), Shanghai Science and Technology Committee grants (16JC1420200).

## Conflict of Interest

The authors declare that the research was conducted in the absence of any commercial or financial relationships that could be construed as a potential conflict of interest.
